# Predicting hemorrhagic transformation and its timing from maximum cerebral lesion diameter in nonlacunar ischemic strokes

**DOI:** 10.1002/brb3.1497

**Published:** 2019-12-17

**Authors:** Antonio Muscari, Luca Faccioli, Maria Vittoria Lega, Andrea Lorusso, Marco Masetti, Marco Pastore Trossello, Giovanni M. Puddu, Luca Spinardi, Marco Zoli

**Affiliations:** ^1^ Stroke Unit Medical Department of Continuity of Care and Disability S.Orsola‐Malpighi Hospital Bologna Italy; ^2^ Department of Medical and Surgical Sciences University of Bologna Bologna Italy; ^3^ Diagnostic and Interventional Neuroradiology Unit S.Orsola‐Malpighi Hospital Bologna Italy

**Keywords:** cerebral lesion diameter, determinants, hemoglobin, hemorrhagic transformation, ischemic stroke, oral anticoagulation

## Abstract

**Objectives:**

We performed this retrospective cohort study to establish which factors are mostly indicative of the appearance of hemorrhagic transformation (HT) and of its time course in a sample of nonlacunar ischemic strokes.

**Materials and Methods:**

In 402 patients with nonlacunar ischemic stroke (75.0 ± 12.7 years, 192 male), clinical, laboratory, and neuroimaging variables obtained during the first 3 days of hospitalization were compared between patients with and without HT at computer tomography scan.

**Results:**

HT was documented in 129 patients (32.1%), including 36 with parenchymal hematoma (PH), after a median time of 6 days (range 1–27). Many variables were univariately associated with HT, but only 5 of them were confirmed in Cox regression (Hazard Ratio, 95% Confidence Interval): maximum cerebral lesion diameter (CLD) in cm (1.12, 1.06–1.18; *p* = .0001), hemoglobin in g/dl (1.16, 1.06–1.27; *p* = .002), blood glucose in mmol/L (1.10, 1.03–1.18; *p* = .007), prior anticoagulant therapy (1.82, 1.10–3.03; *p* = .02), and edema with mass effect (1.72, 1.08–2.75; *p* = .02). Thus, the most significant predictor was CLD. The overall risk of HT was minimum for CLD < 2 cm (1.5%), intermediate for CLD ≥ 2 and < 5 cm (22%), and maximum for CLD ≥ 5 cm (58%). The residual probability of having HT decreased progressively over time, and a simple formula is proposed to predict, from CLD in cm, when the probability of HT falls below 10%.

**Conclusions:**

The main determinant of HT was CLD, a simple quantitative parameter that could prove useful, in particular, in deciding the timing of anticoagulation in cardioembolic stroke patients.

## Graphical abstract

In our sample of 402 nonlacunar ischemic strokes, the main predictors of hemorrhagic transformation were as follows: (a) cerebral lesion diameter, (b) blood glucose, (c) prior anticoagulant therapy, (d) cerebral edema with mass effect, (e) hemoglobin concentration. A simple relationship between cerebral lesion diameter and the timing of hemorrhagic transformation has been found, which could be considered in deciding when starting anticoagulation after a cardioembolic stroke.

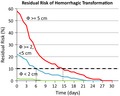

## 1 INTRODUCTION

One of the main unfavorable evolutions of cerebral infarct is hemorrhagic transformation (HT). It is a relatively frequent event (on average 8.5% of the cases, ranging from 0% to 85% in different studies [Lindley et al., 2004]). Its clinical manifestations may vary from total absence of symptoms (mild “hemorrhagic infarct”), up to the progression of neurological deficit and patient's death (severe “parenchymal hematoma”).

In patients with cardioembolic stroke, the assessment of HT risk is particularly relevant: even if no signs of bleeding are present at neuroimages, early anticoagulation in high‐risk patients might favor subsequent important bleedings (Coull et al., 2002; Marsh, Llinas, Hillis, & Gottesman, 2013). Vice versa, delaying the treatment would uselessly expose low‐risk subjects to increased cardioembolic risk. Many studies have dealt with this topic, with particular reference to the identification of the factors that may suggest avoiding thrombolysis (Strbian et al., 2014). In this case, the risk indicators should be immediately available on hospital admission. Instead, in view of deciding when starting oral anticoagulation, the risk indicators may be obtained during the first 3–4 days, as current guidelines suggest that before that time anticoagulation would be hazardous (Kirchhof et al., 2016; Paciaroni et al., 2015, 2017; Powers et al., 2018).

The proposed risk indicators have been numerous. Foremost, cerebral infarct size (Kerenyi et al., 2006; Lee et al., 2010; Marsh et al., 2016; Paciaroni et al., 2008) and, in addition, diabetes and hyperglycemia (Kerenyi et al., 2006; Kunte et al., 2012; Marsh et al., 2016; Paciaroni et al., 2009, 2008; Wang, Yang, Lin, & Lu, 2014), age (Kunte et al., 2012; Larrue, Kummer, Zoppo, & Bluhmki, 1997; Marsh et al., 2016; Paciaroni et al., 2009; Wang et al., 2014), male sex (Ögren, Irewall, Bergström, & Mooe, 2015), female sex (Chen et al., 2016), cardioembolic stroke (Chen et al., 2016; Paciaroni et al., 2008; Wang et al., 2014), thrombolysis (Kablau et al., 2011; Larrue et al., 1997; Ögren et al., 2015; Paciaroni et al., 2008), National Institutes of Health Stroke Scale (NIHSS) score (Guo, Wu, Zhang, Mikulis, & terBrugger, 2006; Wang et al., 2014), previous hemorrhagic stroke (Lee et al., 2010; Ögren et al., 2015), renal insufficiency with reduced glomerular filtration rate (Lee et al., 2013; Marsh et al., 2016), oral anticoagulant therapy (Marsh et al., 2016), hyperintense appearance of middle cerebral artery at neuroimages (Guo et al., 2006), low‐density lipoprotein hypocholesterolemia (D'Amelio et al., 2011; Wang et al., 2014), leukocytosis (Marsh et al., 2016), low platelet count (Lee et al., 2010), and high C‐reactive protein (Lee et al., 2010).

Despite this plethora of possible indicators of HT probability, the attempts to practically utilize these items of information have been rather scanty and not easily applicable in the clinical setting. For example, Marsh et al. (2013) have proposed three parameters (age, cerebral infarct volume, and glomerular filtration rate) which, within the logistic equation, allow the calculation of the probability of HT (HeRS score). In a validation study (Marsh et al., 2016), the same authors decided to add three further parameters (serum glucose, white blood cell count, and warfarin use prior to admission) to obtain a satisfactory area under the ROC curve. However, the difficulty of measuring infarct volume and calculating HT probability by the logistic equation makes this score hardly applicable in the clinical routine. More recently, Kalinin, Khasanova, and Ibatullin (2017) have proposed a simple scoring system, the Hemorrhagic Transformation Index (HTI), to identify acute ischemic stroke patients at high risk of having HT during the first 14 days of hospitalization (their sample also included lacunar strokes). The index is derived from four items: Alberta Stroke Program Early CT (ASPECT) score, initial NIHSS score, hyperdense middle cerebral artery sign, and atrial fibrillation on admission. The authors reported very good HT predictivity with their index, but they did not provide any indication on how this information should be used to decide the timing of anticoagulation in cardioembolic strokes. In addition, their index can be applied only to strokes in the middle cerebral artery territory.

Based on these premises, we performed a retrospective study in the hypothesis that one or more of the previously assessed variables, or some new factor, may be indicative of the appearance of HT and of its time course in nonlacunar ischemic strokes located in any vascular territory. In particular, we utilized a simple quantitative method to measure infarct size, namely its maximum diameter in cm. In addition, we propose a predictive method, easily applicable at patient's bed, which might prove useful in deciding the timing of anticoagulation in cardioembolic strokes.

## 2 MATERIALS AND METHODS

### 2.1 Patients

**Figure 1:**
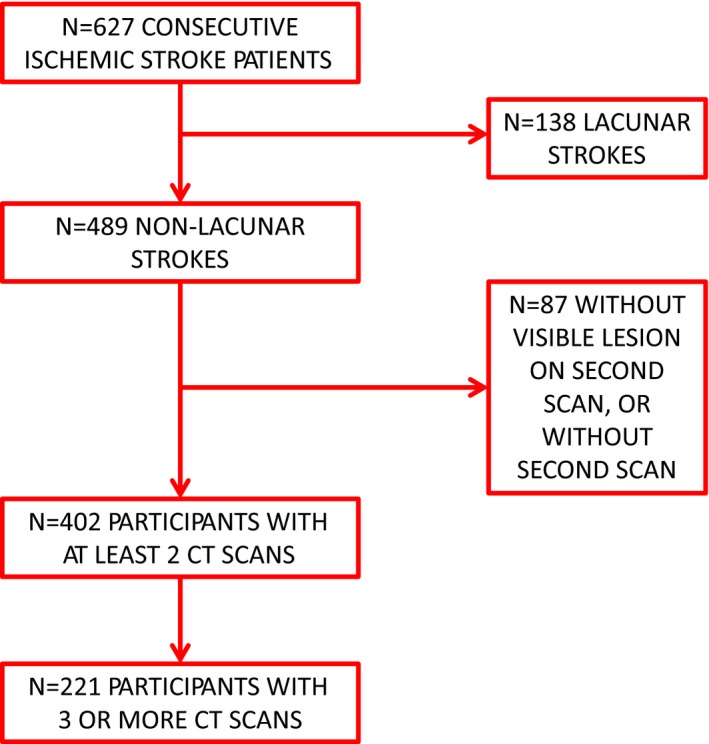
Flow diagram of patient selection and CT scan execution

We considered for inclusion in the study 627 patients with ischemic stroke consecutively admitted to our stroke unit from February 2011 to January 2014, within 24 hr from the onset of symptoms. Ischemic stroke was defined as a sudden and persistent focal neurological deficit not associated with signs of cerebral hemorrhage on first CT scan. Lacunar strokes are associated with ischemic lesions of small size that usually do not present HT. Moreover, since by definition the strokes originated in a small artery are not deemed cardioembolic, they do not need any anticoagulant therapy. For these reasons, small artery strokes, defined according to the Trial of ORG 10,172 in Acute Stroke Treatment (TOAST) criteria (Adams et al., 1993), were excluded from the study (*N* = 138). In addition, further cases of ischemic stroke without 2 CT scans performed, or without any visible cerebral lesion on second CT scan, were also excluded (*N* = 87). Thus, the participants were 402 (mean age 75.0 ± 12.9 years; 192 male), including cases of ischemic stroke from large artery, cardioembolism, other determined cause, or undetermined cause according to the TOAST classification (Figure 1 and Table 1).

**Table 1:** Baseline clinical variables and risk factors in patients with and without hemorrhagic transformation of cerebral lesion

Baseline variable	No HT (*N* = 273)	Any HT (*N* = 129)	*p* Value^a^	PH (*N* = 36)	*p* Value^b^
Age (years)	75.1 ± 13.3	74.1 ± 12.9	.45	72.5 ± 10.3	.17
Male sex	123 (45.1)	69 (53.5)	.11	22 (61.1)	.07
OCSP classification					
LACS	5 (1.8)	2 (1.6)	.84	0	.41
PACS	150 (54.9)	34 (26.4)	<.0001	8 (22.2)	.0002
TACS	76 (27.8)	78 (60.5)	<.0001	26 (72.2)	<.0001
POCS	42 (15.4)	15 (11.6)	.31	2 (5.6)	.11
TOAST classification					
Large artery	64 (23.4)	18 (14.0)	.03	3 (8.3)	.04
Cardioembolism	101 (37.0)	67 (51.9)	.005	18 (50.0)	.13
Other determined cause	14 (5.1)	2 (1.6)	.09	0	.16
Undetermined cause	94 (34.4)	42 (32.6)	.71	15 (41.7)	.39
NIHSS score	8 [4–15]	16 [8–20]	<.0001	16.5 [13.5–21]	<.0001
GCS score	15 [13–15]	13 [10–15]	<.0001	13 [10–14]	.0003
Hypertension	219 (80.2)	111 (86.0)	.15	31 (86.1)	.40
Ex‐smoker	54 (19.8)	32 (24.8)	.25	10 (27.8)	.27
Current smoker	56 (20.5)	27 (20.9)	.92	10 (27.8)	.32
Diabetes	51 (18.7)	28 (21.7)	.48	4 (11.1)	.26
Hypercholesterolemia	154 (56.4)	81 (62.8)	.23	23 (63.9)	.39
Alcohol	25 (9.2)	12 (9.3)	.96	4 (11.1)	.71
Atrial fibrillation	108 (39.6)	74 (57.4)	.0008	22 (61.1)	.01
Previous ischemic stroke	32 (11.7)	17 (13.2)	.68	1 (2.8)	.10
Previous TIA	15 (5.5)	7 (5.4)	.98	0	.15
Previous hemorrhagic stroke	5 (1.8)	3 (2.3)	.74	1 (2.8)	.70
Previous myocardial infarction	35 (12.8)	21 (16.3)	.35	8 (22.2)	.13
Heart failure	26 (9.5)	10 (7.8)	.56	4 (11.1)	.76
Chronic kidney disease	23 (8.4)	13 (10.1)	.59	3 (8.3)	.99
Preadmission antiplatelet drug	120 (44.0)	61 (47.3)	.53	18 (50.0)	.49
Preadmission oral anticoagulant	20 (7.3)	20 (15.5)	.01	6 (16.7)	.06
Average SBP (mmHg)	140.2 ± 20.9	139.4 ± 19.1	.74	141.4 ± 20.6	.73
Average DBP (mmHg)	75.3 ± 10.1	76.2 ± 9.1	.42	75.5 ± 10.7	.94
Average heart rate (beats/min)	75.8 ± 13.2	76.5 ± 14.7	.65	74.7 ± 15.2	.62
Maximum body temperature (°C)	36.8 [36.0–37.4]	37.2 [36.7–37.9]	.0001	38.0 [37.0–38.1]	<.0001
Thrombolysis	35 (12.8)	19 (14.7)	.60	8 (22.2)	.13
In‐hospital antiplatelet drug	260 (95.2)	108 (83.7)	.0001	23 (63.9)	<.0001
In‐hospital low‐dose subcutaneous heparin	181 (66.3)	104 (80.6)	.003	26 (72.2)	.48
In‐hospital oral anticoagulant	81 (29.7)	6 (4.7)	<.0001	2 (5.6)	.002

Note

Numeric data are mean ± *SD*, or median [25th–75th percentile], or number (percentage).

Abbreviations: DBP, diastolic blood pressure; GCS, Glasgow Coma Scale; HT, Hemorrhagic transformation; LACS, lacunar circulation syndrome; NIHSS, National Institutes of Health Stroke Scale; OCSP, Oxfordshire Community Stroke Project; PACS, partial anterior circulation syndrome; PH, Parenchymal hematoma; POCS, posterior circulation syndrome; SBP, systolic blood pressure; TACS, total anterior circulation syndrome; TIA, transient ischemic attack; TOAST, Trial of ORG 10172 in Acute Stroke Treatment.

a Any HT vs. No HT.

b PH vs. No HT.

Because of the retrospective nature of this study, which included several patients deceased after the stroke, a written informed consent could not be obtained. However, the utilization for the present study of the data derived from medical records was approved by our joint university‐hospital Ethics Committee, and the study was conducted in accordance with the WMA Declaration of Helsinki.

Aim of the study was the identification of HT determinants among a series of clinical, laboratory, and instrumental variables obtained during the first 3 days of stay (or, in patients with earlier HT, before HT detection). The patients under antihypertensive treatment, or with mean systolic blood pressure ≥ 140 mmHg or mean diastolic blood pressure ≥ 90 mmHg during the first 3 days of hospitalization, were considered hypertensive. The patients under antidiabetic treatment, or with fasting blood glucose ≥ 7 mmol/L on the day after admission, were considered diabetic. The patients under statin treatment, or with serum total cholesterol ≥ 5.18 mmol/L, were considered hypercholesterolemic. The patients who referred to drink any amount of alcohol in a nonoccasional manner were considered alcohol drinkers. The mean heart rate and systolic and diastolic blood pressure were calculated from the values obtained in the morning during the first 3 days. The maximum body temperature in °C during the first 3 days was also recorded (tympanic temperature was measured 3 times a day). Furthermore, previous major ischemic events, the occurrence of current or previous atrial fibrillation, treatment with antiplatelet or anticoagulant drugs prior to admission, and treatment with i.v. thrombolysis were recorded.

Stroke severity was assessed on admission by the NIHSS score (Lyden et al., 1999) and the Oxfordshire Community Stroke Project classification (Bamford, Sandercock, Dennis, Burn, & Warlow, 1991). The impairment of consciousness was assessed on admission by the Glasgow Coma Scale (Teasdale & Jennett, 1974). Finally, the etiology of stroke was established according to the TOAST criteria (Adams et al., 1993).

### 2.2 Brain CT scan execution and assessment

In our hospital, routine neuroimaging for stroke is normally obtained by noncontrast CT scans, as suggested by current guidelines (Powers et al., 2018). Brain CT scans were performed by a LightSpeed scanner (General Electric Medical Systems), and the measurements on CT scans were performed by Kodak Carestream PACS Web Software (Eastman Kodak Company).

All patients (*N* = 402) underwent their first CT scan on admission in the emergency department (day 0). In addition, all of them underwent a second scan (median time: day 3 after admission, interquartile range 2–6), to visualize cerebral lesion and possible HT. Finally, 221 patients (55%) underwent 3 or more scans after a median time of 13 days (IQR 7–19), mainly to exclude HT in cardioembolic strokes before starting or restarting anticoagulation (at that time the European guidelines suggested waiting 14 days or longer before starting anticoagulation [European Heart Rhythm Association et al., 2010]; Figure 1). The median number of CT scans performed was 3 (interquartile range 2–3).

**Figure 2:**
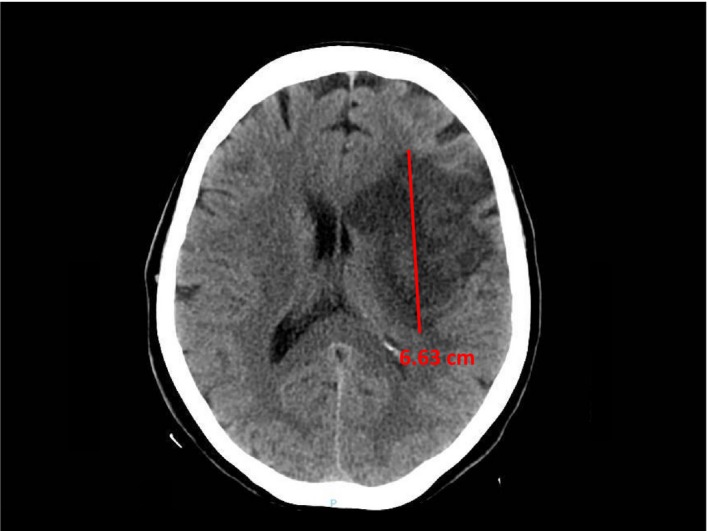
CT image showing an example of maximum cerebral lesion diameter measurement and mass effect

All CT scans were reassessed and agreed by 2 expert neuroradiologists (L.F. and L.S.) who inspected the images together and recorded the following aspects:

1. Hemorrhagic transformation – HT was classified into 4 categories according to the European Cooperative Acute Stroke Study (ECASS) criteria (Fiorelli et al., 1999): HI 1 (hemorrhagic infarct with isolated petechiae), HI 2 (hemorrhagic infarct with confluent petechiae), PH 1 (parenchymal hematoma ≤ 30% of the infarcted area with some mild mass effect), and PH 2 (parenchymal hematoma > 30% of the infarcted area with mass effect usually causing midline shift). These aspects were searched in all CT scans performed during the stay (between 1 and 27 days after admission).
2. Cerebral lesion diameter (CLD) – This was normally obtained on second CT scan (on average 3 days after admission), by measuring the maximum diameter in cm in the CT slice where the maximum extension of the ischemic area was present (Figure 2).
3. Mass effect – Compression of cerebral ventricles and/or subarachnoid spaces, assessed on second CT scan, indicating cerebral edema (Figure 2).
4. White matter lesions – Diffuse white matter hypodensity on first CT scan as expression of chronic cerebral microvascular disease. In this study, white matter lesions were defined as any degree ≥ 1 according to Van Swieten, Hijdra, Koudstaal, and Gijn (1990).
5. Hyperdense appearance of middle cerebral artery, suggesting possible thrombosis, visible on first CT scan.

### 2.3 Laboratory variables

Routine laboratory variables were obtained from the fasting blood sample withdrawn on the first morning after admission (“the baseline”). The laboratory variables obtained on admission were disregarded, as they could potentially be altered by the acute phase.

### 2.4 Statistical analysis

Normal continuous variables were described by mean ± *SD*, while the variables with nongaussian distribution were described by median and interquartile range. Means were compared with Student's *t* test, medians with Mann‐Whitney's test and percentages with chi‐squared test.

Multivariable analyses were performed by Cox proportional hazards regressions with backward stepwise procedure to obtain hazard ratios and 95% confidence intervals, with any HT or PH as dependent variable. This type of regression takes into account the day of appearance of HT/PH. In the cases without HT/PH, the day of last CT scan performed was entered.

For each day between 0 and 30 days after admission, the percent residual probability of HT was calculated as the ratio between the number of patients who had HT from that day onwards and the total number of patients.

Two‐tailed tests were used throughout, and *p* values < .05 were considered significant. Statistical analysis was performed with SPSS software (SYSTAT 10, SPSS Inc).

## 3 RESULTS

Of the 402 patients with nonlacunar stroke included in the study, 129 had HT (32.1%). In particular, 37 had HI 1 (9.2% of 402), 56 HI 2 (13.9%), 23 PH 1 (5.7%), and 13 PH 2 (3.2%). HT was detected after a median time of 6 days (interquartile range 4–11, overall range 1–27).

As shown in Table 1, the patients with any HT, compared with the patients without HT, had more severe strokes, characterized by higher NIHSS and lower GCS scores, and higher prevalence of total anterior circulation syndromes. For completeness, also the TOAST classification is shown, although in some cases, it could be established after the third day of stay. Seven patients (2 with HT and 5 without) were clinically classified as lacunar circulation syndromes (LACS), but subsequently, they were found to have nonlacunar cortical lesions. Other HT determinants were cardioembolism, high body temperature, and prior oral anticoagulant therapy. Instead, cardiovascular risk factors (including age and sex), hemodynamic parameters, previous cerebrovascular events, and preadmission antiplatelet treatment were not associated with HT. The same associations, except those concerning cardioembolism and prior oral anticoagulation, were confirmed in the subgroup with PH 1 or 2. The table also reports the in‐hospital administration of anticoagulants, antiplatelet drugs, and subcutaneous heparin before HT detection or, for patients without HT, during the whole stay in stroke unit. Eighty‐seven patients were treated with oral anticoagulants (mainly cardioembolic strokes). Among them, only in 6 cases oral anticoagulation preceded HT detection. Subcutaneous heparin was more often prescribed to patients with HT, while antiplatelet drugs were more often prescribed to patients without HT. In fact, many patients had HT on the first day, associated with paralysis of a lower limb. For them, subcutaneous heparin was mandatory, while antiplatelet drug prescription could be postponed (particularly in case of PH) to avoid worsening of HT.

In addition (Table 2), the patients with any HT or PH had higher baseline values of white blood cells, hemoglobin, hematocrit, C‐reactive protein, blood glucose and lower levels of HDL cholesterol (except PH patients), and, more frequently, a hyperdense appearance of middle cerebral artery. Finally, they also had larger ischemic lesions and more often mass effect.

**Table 2:** Laboratory and neuroradiologic variables in patients with and without hemorrhagic transformation of cerebral lesion

Baseline variable	No HT (*N* = 273)	Any HT (*N* = 129)	*p* Value^a^	PH (*N* = 36)	*p* Value^b^
INR	1.07 [1.02–1.13]	1.09 [1.04–1.17]	.06	1.09 [1.04–1.14]	.21
aPTT (ratio)	0.94 [0.85–1.06]	0.94 [0.84–1.04]	.41	0.92 [0.84–1.01]	.40
White blood cells (×10^9^/L)	8.68 [6.96–10.60]	9.71 [7.97–11.24]	.0008	10.47 [9.03–12.87]	.0001
Hemoglobin (g/dl)	13.4 [11.9–14.5]	13.9 [12.6–14.8]	.004	14.5 [12.4–15.3]	.01
Hematocrit (%)	40.4 [36.1–44.0]	42.1 [39.1–44.8]	.0007	44.1 [38.1–45.7]	.006
Platelet count (×10^9^/L)	243.9 [183–277]	217.5 [178.5–280.0]	.38	200.5 [167.0–242.0]	.08
C‐reactive protein (mg/dl)	0.81 [0.34–3.24]	1.45 [0.51–3.66]	.02	3.27 [0.74–5.51]	.004
Blood glucose (mmol/L)	5.16 [4.55–6.11]	5.88 [5.05–7.05]	<.0001	6.05 [5.16–6.99]	.006
Cholesterol (mmol/L)	5.04 ± 0.41	4.91 ± 1.13	.28	4.80 ± 0.96	.23
HDL Cholesterol (mmol/L)	1.37 ± 0.41	1.27 ± 0.35	.02	1.31 ± 0.33	.36
Triglycerides (mmol/L)	1.17 [0.93–1.53]	1.16 [0.88–1.44]	.51	1.10 [0.80–1.42]	.35
Creatinine (μmol/L)	78.7 [68.1–95.0]	77.8 [68.1–92.8]	.88	82.2 [69.0–88.4]	.81
White matter lesions	86 (31.5)	34 (26.4)	.29	7 (19.4)	.14
Hyperdense MCA sign	42 (15.4)	47 (36.4)	<.0001	12 (33.3)	.008
Cerebral lesion diameter (cm)	3.3 [2.1–4.7]	6.2 [4.4–9.4]	<.0001	7.0 [5.9–10.3]	<.0001
Edema with mass effect	107 (39.2)	92 (71.3)	<.0001	33 (91.7)	<.0001

Note

Numeric data are mean ± *SD*, or median [25th–75th percentile], or number (percentage).

Abbreviations: HDL, high density lipoprotein; HT, hemorrhagic transformation; INR, international normalized ratio; MCA, middle cerebral artery; PH, parenchymal hematoma.

a Any HT vs. No HT.

b PH vs. No HT.

The variables associated with HT or PH with *p* values < .05 were included as independent variables in two Cox regressions, where the dependent variables were, respectively, HT and PH (Table 3). In these analyses, the patients with missing data were excluded. In addition, the analysis with PH as dependent variable did not include the patients with HI. At the end of the procedures, four variables remained significantly associated with both HT and PH: CLD, hemoglobin concentration (which was missing in two non‐HT patients), blood glucose (missing in six non‐HT and three HT patients), and mass effect. Preadmission anticoagulant therapy remained associated with HT only.

**Table 3:** Baseline variables independently associated with the appearance of hemorrhagic transformation

Variable	Hazard ratio	95% Confidence interval	*p* Value
Dependent variable: Any Hemorrhagic Transformation (*N* = 117/363)^a^			
Cerebral lesion diameter (cm)	1.12	1.06–1.18	.0001
Hemoglobin (g/dL)	1.16	1.06–1.27	.002
Blood glucose (mmol/L)	1.10	1.03–1.18	.007
Preadmission oral anticoagulation	1.82	1.10–3.03	.02
Edema with mass effect	1.72	1.08–2.75	.02
Dependent variable: Parenchymal Hematoma (*N* = 36/282)^b^			
Edema with mass effect	7.84	2.26–27.23	.001
Cerebral lesion diameter (cm)	1.16	1.05–1.28	.005
Hemoglobin (g/dl)	1.25	1.04–1.51	.02
Blood glucose (mmol/L)	1.13	1.01–1.26	.04

Note

Final result of Cox regressions with backward elimination procedure of nonsignificant associations. The initial models included all variables associated with hemorrhagic transformation or parenchymal hematoma with *p* values < .05 in Tables 1 and 2.

a Patients with missing data were excluded.

b Patients with missing data or hemorrhagic infarct (HI) were excluded.

Table 3 shows that the most significant determinant of any HT was CLD (*p* = .0001), with a 12% increase in HT probability for every cm of diameter. Table 4 shows that the relationship between CLD and frequency of HT was very close and progressive. The table also shows that for each lesion size the cumulative frequency of HT increased in relation to the time of CT execution. Six cases of HT were detected on admission (4.7% of all HTs, including 2 PHs), namely, before CLD and edema assessment and baseline blood sampling. These patients were not excluded, not to alter the complete picture of HT incidence and timing. According to CLD and final HT frequency, the whole sample (*N* = 402) could be divided into three main areas of bleeding risk: low risk (<2 cm, *N* = 65, HT 1.5%, PH 0%), intermediate risk (≥2 and <5 cm, *N* = 187, HT 21.9%, PH 3.7%), and high risk (≥5 cm, *N* = 150, HT 58.0%, PH 19.3%). The limit between intermediate and high risk was set at 5 cm because over that limit the probability of HT exceeded 50%. The six cases of HT detected after oral anticoagulation were so distributed: none among low‐risk patients, two among intermediate‐risk patients, and four among high‐risk patients.

**Table 4:** Frequency of hemorrhagic transformation in relation to cerebral lesion diameter and CT scan timing

Lesion (cm)	*N*	1st CT	Up to 2nd CT	Up to 3rd or further CT		
		HT	HT	HT	PH	Overall risk
≥9	57	2 (3.5%)	24 (42.1%)	37 (64.9%)	13 (22.8%)	HIGH
≥7 (<9)	33	1 (3.0%)	12 (36.4%)	19 (57.6%)	5 (15.2%)	HT = 87/150 = 58.0%
≥5 (<7)	60	1 (1.7%)	17 (28.3%)	31 (51.7%)	11 (18.3%)	PH = 29/150 = 19.3%
≥4 (<5)	58	2 (3.4%)	8 (13.8%)	18 (31.0%)	3 (5.2%)	INTERMEDIATE
≥3 (<4)	70	0	10 (14.3%)	15 (21.4%)	1 (1.4%)	HT = 41/187 = 21.9%
≥2 (<3)	59	0	5 (8.5%)	8 (13.6%)	3 (3.4%)	PH = 7/187 = 3.7%
>0 (<2)	65	0	1 (1.5%)	1 (1.5%)	0	LOW HT = 1/65 = 1.5% PH = 0/65 = 0%

Note

1st CT scan: on admission (402 patients); 2nd CT scan: on average on day 3 (402 patients); and further CT scans: on average on day 13 (221 patients).

**Figure 3:**
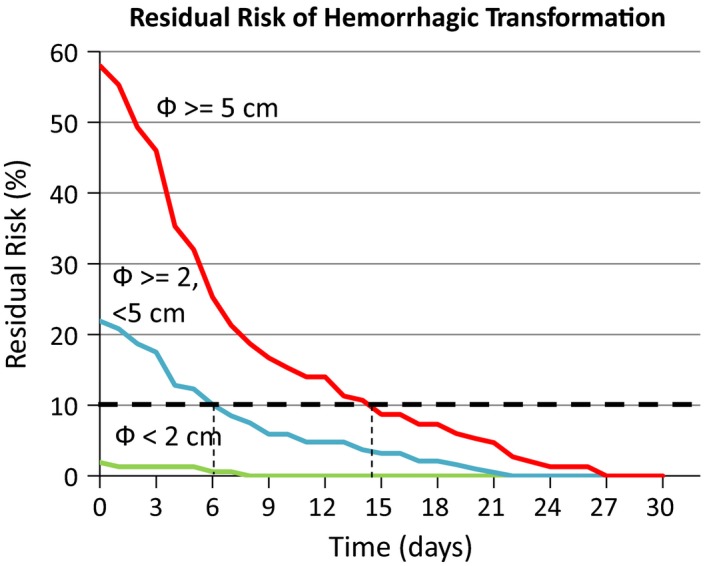
Residual risk of having any hemorrhagic transformation in relation to time and cerebral lesion diameter. The percentage of patients that could have hemorrhagic transformation dropped below 10% after the 6th day with cerebral lesion diameters from 2 to <5 cm, or after the 14th day with cerebral lesion diameters equal or greater than 5 cm

Figure 3 shows the residual probability of having any HT in relation to time in the three areas of risk. Most HTs occurred during the first days and, in each area of risk, the percentage of patients that could have HT decreased progressively over time. This percentage fell below 10% after the 6th day for patients with CLD from 2 to <5 cm or after the 14th day for patients with CLD equal or greater than 5 cm. The figure is based on all available CT scans, that is, at least 2 scans for all patients and 3 or more scans for 221 patients. The patients with 3 or more scans were so distributed in the 3 areas of risk: 37 (56.9%) among low‐risk patients, 94 (50.3%) among intermediate‐risk patients, and 90 (60.0%) among high‐risk patients (*p* = .19).

This information might prove useful in deciding the timing of anticoagulation in patients with cardioembolic stroke. The decision should also consider the indications of Paciaroni et al. (2015, 2017), according to which the interval of 3–4 to 14 days after stroke onset would be the best one to start anticoagulation. Thus (Table 5), (a) the minimum interval (3–4 days) could be applied to the patients with the minimum risk (lesion < 2 cm, 16% of the patients with nonlacunar stroke); (b) an intermediate interval (6 days, see Figure 3) could be applied to the patients with intermediate‐risk (lesion from 2 to <5 cm, 47% of the patients with nonlacunar stroke); and (c) finally, the maximum interval (14 days, see Figure 3) could be applied to the patients with high risk (lesion ≥ 5 cm, 37% of the patients with nonlacunar stroke). In addition, within the two categories of intermediate and maximum risk, the day on which the probability of HT becomes lower than 10% can be extrapolated in relation to the precise size of cerebral lesion (Table 5). To remember Table 5, we propose this simple formula: number of days from stroke onset = CLD + 3 (if CLD 0–4 cm) or CLD + 7 (if CLD ≥ 5 cm).

**Table 5:** Proposed fractioning of time‐to‐low‐risk of hemorrhagic transformation according to exact lesion size

Overall risk	Mean interval after which residual risk falls below 10% (days)^a^	Lesion (cm)	Proposed time‐to‐low‐risk (days)^a^
HIGH HT = 87/150 = 58.0%	14	≥9	16
		≥7 (<9)	**14**
		≥5 (<7)	12
INTERMEDIATE HT = 41/187 = 21.9%	6	≥4 (<5)	7
		≥3 (<4)	**6**
		≥2 (<3)	5
LOW HT = 1/65 = 1.5%	0	>0 (<2)	**3–4**

The numbers in bold are those experimentally found. The other numbers are extrapolated.

a See Figure 3 and text.

## 4 DISCUSSION

This study has shown that, in patients with nonlacunar ischemic stroke, among numerous variables obtained during the first 3 days of hospitalization, the variable most closely associated with the appearance of HT was the size of cerebral lesion. It seems therefore reasonable to estimate the probability of HT and its timing starting from this parameter.

The frequency of HT in our study (32.1%) was higher than the average frequency reported in the literature (Lindley et al., 2004). This finding is explainable considering that lacunar strokes were excluded from the study and that all the available CT scans were used to detect HT (up to 27 days after the acute event).

It is well known that the size of the ischemic lesion is an important factor favoring HT (Kerenyi et al., 2006; Lee et al., 2010; Marsh et al., 2016; Paciaroni et al., 2008). According to the current European guidelines on atrial fibrillation (Kirchhof et al., 2016), both the size of brain infarction and HT should be taken into account to decide when starting anticoagulation. However, the same guidelines only generically suggest “delaying” oral anticoagulation in the presence of “large/moderate” lesions, without specifying how long the delay should be and what large/moderate means. In addition, NIHSS score is used as primary indicator of the timing of anticoagulation. NIHSS score is correlated, in principle, with the extension of the ischemic lesion. However, in some cases, low NIHSS scores may be associated with large lesions (nondominant hemisphere, occipital lobe, and cerebellum), while on the contrary, there may be small lesions causing elevated NIHSS scores (internal capsule and brainstem). And, in fact, in our multivariable analysis, which also included CLD, NIHSS was eliminated. Moreover, several other factors (e.g., mechanical valve prosthesis, severe uncontrolled hypertension, or other determinants evidenced in this study) should be considered in deciding whether to anticipate or delay the timing of anticoagulation.

If CLD has to be used as one possible factor indicating the timing of anticoagulation, we should remember that the ischemic lesion is clearly visible on CT scan only after 24–72 hr from the onset of symptoms. This delay would be, however, compatible with the fact that initiating anticoagulation earlier than 3–4 days would be hazardous according to Paciaroni et al. (2015). A previous study from the VISTA registry (Abdul‐Rahim et al., 2015) suggested that anticoagulation could be performed safely even earlier. However, the authors recognized that the highest risk of both stroke recurrence and symptomatic intracerebral hemorrhage was in the first 2 days. Nonvitamin K antagonist oral anticoagulants (NOACs) are associated with a lower risk of intracranial hemorrhage than vitamin K antagonists. Thus, they might be prescribed earlier after an acute stroke. This possibility is currently being assessed in three different randomized trials (ELAN, START, and TIMING; Åsberg et al., 2017; Ng & Whiteley, 2017). However, a large registry study on the same subject (Paciaroni et al., 2017) recently showed that the minimum composite rate of recurrence and major bleeding was obtained, again, when NOACs were started between 3 and 14 days after acute stroke.

After literature revision, Paciaroni, Agnelli, Ageno, and Caso (2016) have proposed an assessment of the probability of HT based on the size of the ischemic lesion (small, medium, or large), considering small infarcts those <1.5 cm, medium infarcts those caused by partial occlusion of cerebral arteries, and large infarcts those caused by complete occlusion of cerebral arteries. Other authors have used ischemic lesion volume, rather than its diameter, to assess the probability of HT (Marsh et al., 2013, 2016). This measurement is certainly more precise, but is more complex to perform, and therefore, it is not suitable for routine use. Instead, as we have shown in the present investigation, the simple measurement of CLD may provide useful information about the occurrence and timing of HT in everyday practice.

Usually, edema with mass effect is associated with large ischemic lesions. However, our multivariable analysis showed that mass effect was associated with HT (especially PH) independently from CLD. On the other hand, mass effect may be a consequence, rather than a cause, of PH, as PH itself determines mass effect.

The existence of a direct association between hemoglobin and HT had never been reported previously. It is noteworthy that hemoglobin was the second parameter most strongly associated with HT after CLD. Thus, it seems that polycythemia may favor HT, while anemia would have a protective effect. The reasons for this are presently unknown, although some explanations can be proposed. HT generally occurs during cerebral lesion reperfusion (Fiehler et al., 2005). Ischemia–reperfusion injury is mainly due to an oxidative mechanism (Kaminski, Bonda, Korecki, & Musial, 2002), also favored by reduced shear stress in the ischemic area (Kumar et al., 2018). Ensuing endothelial dysfunction increases vascular permeability (Lum & Roebuck, 2001), with possible blood extravasation (Lin et al., 2007). While hematological disorders and polycythemia are well‐known (although sometimes unrecognized) causes of ischemic stroke (Arboix, Jiménez, Massons, Parra, & Besses, 2016), anemia with reduced blood density might favor the microcirculation, with increased shear stress, less endothelial dysfunction, and reduced permeability of the blood–brain barrier. In addition, the free iron released by erythrocytes exposed to hypoxia‐reoxygenation may contribute to oxidant stress (Ciccoli et al., 2004). Thus, fewer red blood cells could mean less free iron and less oxidant stress in the ischemic area. Finally, ischemic and hypoxic preconditioning has been shown to protect the brain from more severe ischemic injuries (Arboix et al., 2004; Ran, Xu, Lu, Bernaudin, & Sharp, 2005; Steiger & Hänggi, 2007). Anemia might possibly exert a protective preconditioning effect, with reduced occurrence of HT.

Current guidelines do not recommend recoagulation in the acute phase of ischemic stroke, in the patients that were previously on oral anticoagulant therapy. Thus, oral anticoagulation is just withdrawn, and if warfarin was used, complete spontaneous recoagulation occurs after a few days. It is therefore understandable that these patients have an increased risk of HT, especially during the first few days of stay, as already shown by others (Marsh et al., 2016). The finding that hyperglycemia favors HT is also in agreement with several previous studies (Kerenyi et al., 2006; Kunte et al., 2012; Marsh et al., 2016; Paciaroni et al., 2009, 2008; Wang et al., 2014).

The main limitations of this study derive from its retrospective design, particularly from the fact that only the available data were collected and some data (especially radiological) were not obtained after prespecified intervals from stroke onset. However, all patients underwent at least two CT scans, and those who underwent three or more scans were numerous and equally distributed in the three areas of risk. In addition, performing multiple scans at regular intervals in all patients would be complicate and probably unethical.

Six patients had HT detected on first CT scan, before some radiological and laboratory variables could be obtained. These patients were not excluded, not to alter the description of HT incidence and timing in its entirety. It seems improbable, however, that these few cases may have significantly influenced the predictive model.

This observational study has sought HT determinants in a cohort of consecutive patients with ischemic stroke: It was not designed to study in detail the probability of HT associated with any particular event. Some events, like previous hemorrhagic stroke or preadmission anticoagulant therapy in patients with PH, had been few, and this might have caused the nonsignificance of their association with HT. Another limitation comes from the possibility that the therapy administered during the first days of stay may have influenced the results. Finally, these results should be confirmed in a different cohort of patients.

## 5 CONCLUSIONS

Although with limitations, this study has shown that, in patients with nonlacunar ischemic stroke, 4 already known parameters (infarct size, mass effect, hyperglycemia, and preadmission anticoagulant therapy) and one new parameter (hemoglobin concentration) collected during the first 3 days of hospitalization are independently associated with the appearance of hemorrhagic transformation. In particular, we have found a simple relationship linking the most relevant of these parameters, cerebral lesion diameter, to the timing of hemorrhagic transformation, which could be considered in deciding when starting or restarting anticoagulation in cardioembolic strokes.

## CONFLICT OF INTEREST

The authors have no conflict of interest to declare.

## Data Availability

The data that support the findings of this study are available from the corresponding author upon reasonable request.

## References

[brb31497-bib-0001] Abdul‐Rahim, A. H. , Fulton, R. L. , Frank, B. , Tatlisumak, T. , Paciaroni, M. , Caso, V. , … VISTA Collaborators (2015). Association of improved outcome in acute ischaemic stroke patients with atrial fibrillation who receive early antithrombotic therapy: Analysis from VISTA. European Journal of Neurology, 22, 1048–1055. 10.1111/ene.12577 25319957

[brb31497-bib-0002] Adams, H. P., Jr. , Bendixen, B. H. , Kappelle, L. J. , Biller, J. , Love, B. B. , Gordon, D. L. , & Marsh, E. E., 3rd. (1993). Classification of subtype of acute ischemic stroke. Definitions for use in a multicenter clinical trial. TOAST. Trial of Org 10172 in Acute Stroke Treatment. Stroke, 24, 35–41. 7678184 10.1161/01.str.24.1.35

[brb31497-bib-0003] Arboix, A. , Cabeza, N. , García‐Eroles, L. , Massons, J. , Oliveres, M. , Targa, C. , & Balcells, M. (2004). Relevance of transient ischemic attack to early neurological recovery after nonlacunar ischemic stroke. Cerebrovascular Disease, 18, 304–311. 10.1159/000080356 15331877

[brb31497-bib-0004] Arboix, A. , Jiménez, C. , Massons, J. , Parra, O. , & Besses, C. (2016). Hematological disorders: A commonly unrecognized cause of acute stroke. Expert Review of Hematology, 9, 891–901. 10.1080/17474086.2016.1208555 27367035

[brb31497-bib-0005] Åsberg, S. , Hijazi, Z. , Norrving, B. , Terént, A. , Öhagen, P. , & Oldgren, J. (2017). Timing of oral anticoagulant therapy in acute ischemic stroke with atrial fibrillation: Study protocol for a registry‐based randomised controlled trial. Trials, 18, 581 10.1186/s13063-017-2313-2319 29197413 PMC5712199

[brb31497-bib-0006] Bamford, J. , Sandercock, P. , Dennis, M. , Burn, J. , & Warlow, C. (1991). Classification and natural history of clinically identifiable subtypes of cerebral infarction. Lancet, 337, 1521–1526. 10.1016/0140-6736(91)93206-O 1675378

[brb31497-bib-0007] Chen, G. , Wang, A. , Zhao, X. , Wang, C. , Liu, L. , Zheng, H. , … Wang, Y. (2016). Frequency and risk factors of spontaneous hemorrhagic transformation following ischemic stroke on the initial brain CT or MRI: Data from the China National Stroke Registry (CNSR). Neurological Research, 38, 538–544. 10.1080/01616412.2016.1187864 27320249

[brb31497-bib-0008] Ciccoli, L. , Rossi, V. , Leoncini, S. , Signorini, C. , Blanco‐Garcia, J. , Aldinucci, C. , … Comporti, M. (2004). Iron release, superoxide production and binding of autologous IgG to band 3 dimers in newborn and adult erythrocytes exposed to hypoxia and hypoxia‐reoxygenation. Biochimica et Biophysica Acta, 1672, 203–213. 10.1016/j.bbagen.2004.04.003 15182940

[brb31497-bib-0009] Coull, B. M. , Williams, L. S. , Goldstein, L. B. , Meschia, J. F. , Heitzman, D. , Chaturvedi, S. , … American Stroke Association (2002). Anticoagulants and antiplatelet agents in acute ischemic stroke: Report of the Joint Stroke Guideline Development Committee of the American Academy of Neurology and the American Stroke Association (a division of the American Heart Association). Stroke, 33, 1934–1942. 10.1161/01.STR.0000028456.18614.93 12105379

[brb31497-bib-0010] D'Amelio, M. , Terruso, V. , Famoso, G. , Ragonese, P. , Aridon, P. , & Savettieri, G. (2011). Cholesterol levels and risk of hemorrhagic transformation after acute ischemic stroke. Cerebrovascular Disease, 32, 234–238. 10.1159/000329315 21860236

[brb31497-bib-0011] European Heart Rhythm Association , European Association for Cardio‐Thoracic Surgery , Camm, A. J. , Kirchhof, P. , Lip, G. Y. , Schotten, U. , … Rutten, F. H. Guidelines for the management of atrial fibrillation: The Task Force for the Management of Atrial Fibrillation of the European Society of Cardiology (ESC). European Heart Journal. 2010;31:2369–2429. 10.1093/eurheartj/ehq278 20802247

[brb31497-bib-0012] Fiehler, J. , Remmele, C. , Kucinski, T. , Rosenkranz, M. , Thomalla, G. , Weiller, C. , … Röther, J. (2005). Reperfusion after severe local perfusion deficit precedes hemorrhagic transformation: An MRI study in acute stroke patients. Cerebrovascular Disease, 19, 117–124. 10.1159/000083180 15640606

[brb31497-bib-0013] Fiorelli, M. , Bastianello, S. , von Kummer, R. , del Zoppo, G. J. , Larrue, V. , Lesaffre, E. , … Bozzao, L. (1999). Hemorrhagic transformation within 36 hours of a cerebral infarct: Relationships with early clinical deterioration and 3‐month outcome in the European Cooperative Acute Stroke Study I (ECASS I) cohort. Stroke, 30, 2280–2284. 10.1161/01.STR.30.11.2280 10548658

[brb31497-bib-0014] Guo, G. , Wu, R. H. , Zhang, Y. P. , Mikulis, D. J. , & terBrugger, K. (2006). Prediction of hemorrhagic transformation after acute ischemic stroke using hyperintense MCA sign. Conference Proceedings IEEE Engineering in Medicine and Biology Society, 1, 1881–1884. 10.1109/IEMBS.2006.260171 17946485

[brb31497-bib-0015] Kablau, M. , Kreisel, S. H. , Sauer, T. , Binder, J. , Szabo, K. , Hennerici, M. G. , & Kern, R. (2011). Predictors and early outcome of hemorrhagic transformation after acute ischemic stroke. Cerebrovascular Disease, 32, 334–341. 10.1159/000331702 21921596

[brb31497-bib-0016] Kalinin, M. N. , Khasanova, D. R. , & Ibatullin, M. M. (2017). The hemorrhagic transformation index score: A prediction tool in middle cerebral artery ischemic stroke. BMC Neurology, 17(1), 177 10.1186/s12883-017-0958-3 28882130 PMC5590157

[brb31497-bib-0017] Kaminski, K. A. , Bonda, T. A. , Korecki, J. , & Musial, W. J. (2002). Oxidative stress and neutrophil activation–the two keystones of ischemia/reperfusion injury. International Journal of Cardiology, 86, 41–59. 10.1016/S0167-5273(02)00189-4 12243849

[brb31497-bib-0018] Kerenyi, L. , Kardos, L. , Szász, J. , Szatmári, S. , Bereczki, D. , Hegedüs, K. , & Csiba, L. (2006). Factors influencing hemorrhagic transformation in ischemic stroke: A clinicopathological comparison. European Journal of Neurology, 13, 1251–1255. 10.1111/j.1468-1331.2006.01489.x 17038041

[brb31497-bib-0019] Kirchhof, P. , Benussi, S. , Kotecha, D. , Ahlsson, A. , Atar, D. , Casadei, B. , … Zeppenfeld, K. (2016). 2016 ESC Guidelines for the management of atrial fibrillation developed in collaboration with EACTS. European Heart Journal, 37, 2893–3962. 10.1093/eurheartj/ehw210 27567408

[brb31497-bib-0020] Kumar, A. , Hung, O. Y. , Piccinelli, M. , Eshtehardi, P. , Corban, M. T. , Sternheim, D. , … Samady, H. (2018). Low coronary wall shear stress is associated with severe endothelial dysfunction in patients with nonobstructive coronary artery disease. JACC: Cardiovascular Interventions, 11, 2072–2080. 10.1016/j.jcin.2018.07.004 30268874 PMC6217963

[brb31497-bib-0021] Kunte, H. , Busch, M. A. , Trostdorf, K. , Vollnberg, B. , Harms, L. , Mehta, R. I. , … Simard, J. M. (2012). Hemorrhagic transformation of ischemic stroke in diabetics on sulfonylureas. Annals of Neurology, 72, 799–806. 10.1002/ana.23680 23280795 PMC3539226

[brb31497-bib-0022] Larrue, V. , von Kummer, R. , del Zoppo, G. , & Bluhmki, E. (1997). Hemorrhagic transformation in acute ischemic stroke. Potential contributing factors in the European Cooperative Acute Stroke Study. Stroke, 28, 957–960. 10.1161/01.STR.28.5.957 9158632

[brb31497-bib-0023] Lee, J. G. , Lee, K. B. , Jang, I. M. , Roh, H. , Ahn, M. Y. , Woo, H. Y. , & Hwang, H. W. (2013). Low glomerular filtration rate increases hemorrhagic transformation in acute ischemic stroke. Cerebrovascular Disease, 35, 53–59. 10.1159/000345087 23428997

[brb31497-bib-0024] Lee, J. H. , Park, K. Y. , Shin, J. H. , Cha, J. K. , Kim, H. Y. , Kwon, J. H. , … Kwon, S. U. (2010). Symptomatic hemorrhagic transformation and its predictors in acute ischemic stroke with atrial fibrillation. European Neurology, 64, 193–200. 10.1159/000319048 20714158

[brb31497-bib-0025] Lin, K. , Kazmi, K. S. , Law, M. , Babb, J. , Peccerelli, N. , & Pramanik, B. K. (2007). Measuring elevated microvascular permeability and predicting hemorrhagic transformation in acute ischemic stroke using first‐pass dynamic perfusion CT imaging. American Journal of Neuroradiology, 28, 1292–1298. 10.3174/ajnr.A0539 17698530 PMC7977671

[brb31497-bib-0026] Lindley, R. I. , Wardlaw, J. M. , Sandercock, P. A. , Rimdusid, P. , Lewis, S. C. , Signorini, D. F. , & Ricci, S. (2004). Frequency and risk factors for spontaneous hemorrhagic transformation of cerebral infarction. Journal of Stroke and Cerebrovascular Diseases, 13, 235–246. 10.1016/j.jstrokecerebrovasdis.2004.03.003 17903981

[brb31497-bib-0027] Lum, H. , & Roebuck, K. A. (2001). Oxidant stress and endothelial cell dysfunction. American Journal of Physiology. Cell Physiology, 280, C719–C741. 10.1152/ajpcell.2001.280.4.C719 11245588

[brb31497-bib-0028] Lyden, P. , Lu, M. , Jackson, C. , Marler, J. , Kothari, R. , Brott, T. , & Zivin, J. (1999). Underlying structure of the National Institutes of Health Stroke Scale: Results of a factor analysis. NINDS tPA Stroke Trial Investigators. Stroke, 30, 2347–2354. 10.1161/01.STR.30.11.2347 10548669

[brb31497-bib-0029] Marsh, E. B. , Llinas, R. H. , Hillis, A. E. , & Gottesman, R. F. (2013). Hemorrhagic transformation in patients with acute ischaemic stroke and an indication for anticoagulation. European Journal of Neurology, 20, 962–967. 10.1111/ene.12126 23521544 PMC3711260

[brb31497-bib-0030] Marsh, E. B. , Llinas, R. H. , Schneider, A. L. , Hillis, A. E. , Lawrence, E. , Dziedzic, P. , & Gottesman, R. F. (2016). Predicting hemorrhagic transformation of acute ischemic stroke: Prospective validation of the HeRS Score. Medicine, 95(2), e2430 10.1097/MD.0000000000002430 26765425 PMC4718251

[brb31497-bib-0031] Ng, K. K. H. , & Whiteley, W. (2017). Anticoagulation timing for atrial fibrillation in acute ischemic stroke: Time to reopen Pandora's box? JAMA Neurology, 74, 1174–1175. 10.1001/jamaneurol.2017.1919 28892535

[brb31497-bib-0032] Ögren, J. , Irewall, A. L. , Bergström, L. , & Mooe, T. (2015). Intracranial hemorrhage after ischemic stroke: Incidence, time trends, and predictors in a Swedish nationwide cohort of 196 765 patients. Circulation: Cardiovascular Quality and Outcomes, 8, 413–420. 10.1161/CIRCOUTCOMES.114.001606 26152682

[brb31497-bib-0033] Paciaroni, M. , Agnelli, G. , Ageno, W. , & Caso, V. (2016). Timing of anticoagulation therapy in patients with acute ischaemic stroke and atrial fibrillation. Thrombosis and Haemostasis, 116, 410–416. 10.1160/TH16-03-0217 27346426

[brb31497-bib-0034] Paciaroni, M. , Agnelli, G. , Caso, V. , Corea, F. , Ageno, W. , Alberti, A. , … Silvestrelli, G. (2009). Acute hyperglycemia and early hemorrhagic transformation in ischemic stroke. Cerebrovascular Disease, 28, 119–123. 10.1159/000223436 19506370

[brb31497-bib-0035] Paciaroni, M. , Agnelli, G. , Corea, F. , Ageno, W. , Alberti, A. , Lanari, A. , … Silvestrelli, G. (2008). Early hemorrhagic transformation of brain infarction: Rate, predictive factors, and influence on clinical outcome: Results of a prospective multicenter study. Stroke, 39, 2249–2256. 10.1161/STROKEAHA.107.510321 18535273

[brb31497-bib-0036] Paciaroni, M. , Agnelli, G. , Falocci, N. , Caso, V. , Becattini, C. , Marcheselli, S. , … Lees, K. R. (2015). Early recurrence and cerebral bleeding in patients with acute ischemic stroke and atrial fibrillation: Effect of anticoagulation and its timing: The RAF study. Stroke, 46, 2175–2182. 10.1161/STROKEAHA.115.008891 26130094

[brb31497-bib-0037] Paciaroni, M. , Agnelli, G. , Falocci, N. , Tsivgoulis, G. , Vadikolias, K. , Liantinioti, C. , … Caso, V. (2017). Early recurrence and major bleeding in patients with acute ischemic stroke and atrial fibrillation treated with non‐vitamin‐K oral anticoagulants (RAF‐NOACs) study. Journal of the American Heart Association, 6(12), e007034 10.1161/JAHA.117.007034 29220330 PMC5779022

[brb31497-bib-0038] Powers, W. J. , Rabinstein, A. A. , Ackerson, T. , Adeoye, O. M. , Bambakidis, N. C. , Becker, K. , … American Heart Association Stroke Council (2018). Guidelines for the early management of patients with acute ischemic stroke: A guideline for healthcare professionals from the American Heart Association/American Stroke Association. Stroke, 2018(49), e46–e110. 10.1161/STR.0000000000000158 29367334

[brb31497-bib-0039] Ran, R. , Xu, H. , Lu, A. , Bernaudin, M. , & Sharp, F. R. (2005). Hypoxia preconditioning in the brain. Developmental Neuroscience, 27, 87–92. 10.1159/000085979 16046841

[brb31497-bib-0040] Steiger, H. J. , & Hänggi, D. (2007). Ischaemic preconditioning of the brain, mechanisms and applications. Acta Neurochirurgica, 149, 1–10. 10.1007/s00701-006-1057-1 17151832

[brb31497-bib-0041] Strbian, D. , Michel, P. , Seiffge, D. J. , Saver, J. L. , Numminen, H. , Meretoja, A. , … Tatlisumak, T. (2014). Symptomatic intracranial hemorrhage after stroke thrombolysis: Comparison of prediction scores. Stroke, 45, 752–758. 10.1161/STROKEAHA.113.003806 24473180

[brb31497-bib-0042] Teasdale, G. , & Jennett, B. (1974). Assessment of coma and impaired consciousness. A practical scale. Lancet, 2, 81–84. 10.1016/S0140-6736(74)91639-0 4136544

[brb31497-bib-0043] van Swieten, J. C. , Hijdra, A. , Koudstaal, P. J. , & van Gijn, J. (1990). Grading white matter lesions on CT and MRI: A simple scale. Journal of Neurology, Neurosurgery and Psychiatry, 53, 1080–1083. 10.1136/jnnp.53.12.1080 PMC488320 2292703

[brb31497-bib-0044] Wang, B. G. , Yang, N. , Lin, M. , & Lu, B. (2014). Analysis of risk factors of hemorrhagic transformation after acute ischemic stroke: Cerebral microbleeds do not correlate with hemorrhagic transformation. Cell Biochemistry and Biophysics, 70, 135–142. 10.1007/s12013-014-9869-8 24691925

